# Glycolysis-Related Genes Serve as Potential Prognostic Biomarkers in Clear Cell Renal Cell Carcinoma

**DOI:** 10.1155/2021/6699808

**Published:** 2021-01-23

**Authors:** Yan Zhang, Mingying Chen, Meihong Liu, Yingkun Xu, Guangzhen Wu

**Affiliations:** ^1^Department of Clinical Laboratory, The First Affiliated Hospital of Dalian Medical University, Dalian, Liaoning 116011, China; ^2^Department of Clinical Laboratory, The First People's Hospital of Linhai, Taizhou, Zhejiang 317000, China; ^3^Department of Respiratory, The First Affiliated Hospital of Dalian Medical University, Dalian, Liaoning 116011, China; ^4^Department of Urology, Shandong Provincial Hospital, Cheeloo College of Medicine, Shandong University, Jinan, Shandong 250021, China; ^5^Department of Urology, The First Affiliated Hospital of Dalian Medical University, Dalian, Liaoning 116011, China

## Abstract

Metabolic rearrangement is a marker of cancer that has been widely studied in recent years. One of the major metabolic characteristics of tumor cells is the high levels of glycolysis, even under aerobic conditions, a phenomenon that is called the “Warburg effect.” We investigated the expression and copy number variation (CNV) frequency of all glycolysis-related genes in multiple cancer types and found many differentially expressed genes, particularly in clear cell renal cell carcinoma (ccRCC). Single nucleotide variants (SNVs) showed that the overall average mutation frequency for all genes was low. The purpose of this study was to establish a predictive model by studying glycolysis-related genes in ccRCC. We compared the expression of glycolysis-related genes in 539 ccRCC tissues and 72 normal renal tissues from The Cancer Genome Atlas dataset and identified 17 upregulated and 26 downregulated genes. Pathway analysis revealed that PSAT1 and SDHB could activate the cell cycle, RPIA could activate the DNA damage response, and HK3 could activate apoptosis and EMT signaling, while PDK2 could inhibit apoptosis. The results of the drug sensitivity analysis suggested that some of these differentially expressed genes were positively correlated with drug sensitivity. Thirteen genes were selected from the gene coexpression network and the LASSO regression analysis. The Kaplan-Meier overall survival curves showed that the expression of upregulated genes in ccRCC patients was associated with lower overall survival. We established a predictive model consisting of 13 genes (RPIA, G6PD, PSAT1, ENO2, HK3, IDH1, PDK4, PGM2, PGK1, FBP1, OGDH, SUCLA2, and SUCLG2). This predictive model correlated well with the development and progression of ccRCC. Thus, it is of great value in the diagnosis and prognostic evaluation of ccRCC and may aid the identification of potential prognostic biomarkers and drug targets.

## 1. Introduction

Renal cell carcinoma (RCC) is the third most common malignant cancer of the urinary system and accounts for approximately 90% of all malignant renal tumors [1, 2]. According to recent statistics, more than 400,000 new cases of RCC have been reported, resulting in approximately 175,000 deaths worldwide annually [3]. In the United States alone, 73,820 new cases of RCC are reported each year and 14,770 people die from this disease [4]. Clear cell renal cell carcinoma (ccRCC) is the most common and fatal subtype, accounting for 75% of all RCCs [5]. As ccRCC is prone to metastasis in the early stages, approximately one-third of the patients exhibit distant metastases at the time of diagnosis [6]. These patients may have missed the best window for treatment. In addition, ccRCC is resistant to radiotherapy and traditional chemotherapy and has a poor prognosis.

For ccRCC, the gold standards for clinical diagnosis include computerized tomography (CT) and histopathological analyses. During the follow-up, CT is routinely performed to gauge disease progression, but histopathological analysis is an invasive procedure, which is not easily accepted by patients and is not suitable for regular monitoring. Moreover, it is difficult to detect small pathological changes in the early stages using CT scans, and there is a risk of radiation damage in patients who need long-term monitoring. At present, the identification of novel diagnostic and prognostic markers for ccRCC is a necessity.

In the early 1920s, Otto Warburg found that even with sufficient oxygen supply, cancer cells converted glucose into lactate [7]. Later, researchers termed this phenomenon as “aerobic glycolysis” or the “Warburg effect” [8]. The Warburg effect is frequently observed in tumors; it is one of the key mechanisms underlying carcinogenesis, and many studies have investigated tumor-related glycolytic genes in recent years [9, 10]. To date, the Warburg effect and the glycolytic genes have been studied in lung cancer, breast cancer, ovarian cancer, gastric cancer, liver cancer, colorectal cancer, esophageal cancer, prostate cancer, bladder cancer, and other tumors [11–19], but there are few reports on ccRCC.

We used bioinformatics to explore glycolytic genes that are differentially expressed in 539 ccRCC tissues and 72 normal control tissues from The Cancer Genome Atlas (TCGA). A predictive model containing 13 genes was established after performing a series of investigations, including pathway analysis, drug sensitivity analysis, gene coexpression analysis, Lasso regression analysis, and survival curve analysis. This model showed great promise for the diagnosis and prognosis of ccRCC and may help in the identification of potential prognostic biomarkers and drug targets in ccRCC.

## 2. Materials and Methods

### 2.1. Data Collection

We acquired the data, including CNV, SNV, and mRNA expression for ccRCC patients, from The Cancer Genome Atlas Database (TCGA) (https://cancergenome.nih.gov/) [20]. TCGA dataset contained 539 ccRCC tissue samples and 72 normal control samples. In addition, we screened 53 glycolysis-related genes based on a literature survey [21].

### 2.2. Pathway Analysis

We analyzed the activation and inhibition of pathways using GSCALite (http://bioinfo.life.hust.edu.cn/web/GSCALite/) [22], which enables the analysis of the following: differential expression between tumor and normal tissues, survival between tumor and normal tissues, pathways related to gene expression, miRNA gene regulatory networks, and drug sensitivity.

### 2.3. Gene Coexpression Network and Transcriptional Factor Network

The gene coexpression network of glycolysis-related genes and heat maps of transcriptional factors were generated using R. We used the Corrplot software package for gene coexpression analysis and the Gene Expression Profiling Interactive Analysis (GEPIA) (http://gepia.cancer-pku.cn/) [23] for analyzing the correlation between different genes. The overall survival (OS) and disease-free survival (DFS) of ccRCC patients were collected from the GEPIA website. Cytoscape was used to visualize the networks of coexpressed genes and transcriptional factors and identified differentially expressed genes [24].

### 2.4. Data Processing and Analysis

We downloaded the official R software from the CRAN website (https://www.r-project.org/). As the R software is complex to navigate, we used RStudio, which is a simple and powerful operation platform (https://www.rstudio.com/). In terms of data processing and analysis, Perl and other R packages were used in this study. The heat maps were generated using Phatmap. The Limma software package was used to perform differential expression analysis of glycolysis-related genes and transcriptional factors. Glmnet and Survival software packages were used to perform the LASSO regression analysis. The survival curves were generated using the Survival software package, and the ROC curves were analyzed and drawn using the survival ROC software package. A *p* < 0.05 indicated a significant difference between the two groups of data.

## 3. Results

### 3.1. Identification of Glycolysis-Related Genes in ccRCC

**Figure 1:**
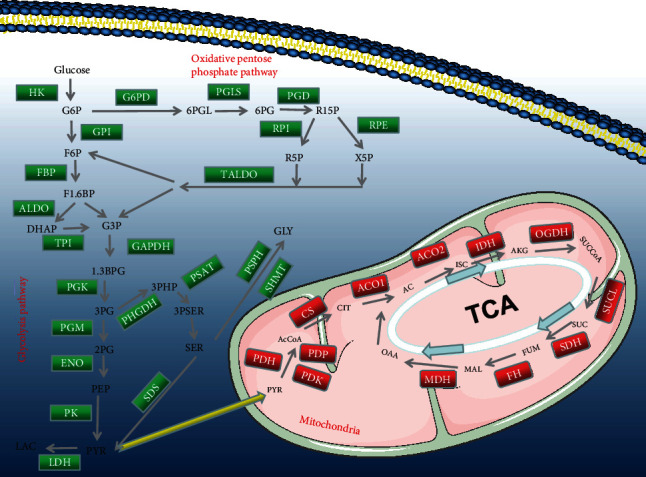
All genes that are involved in the glycometabolism.

**Figure 2:**
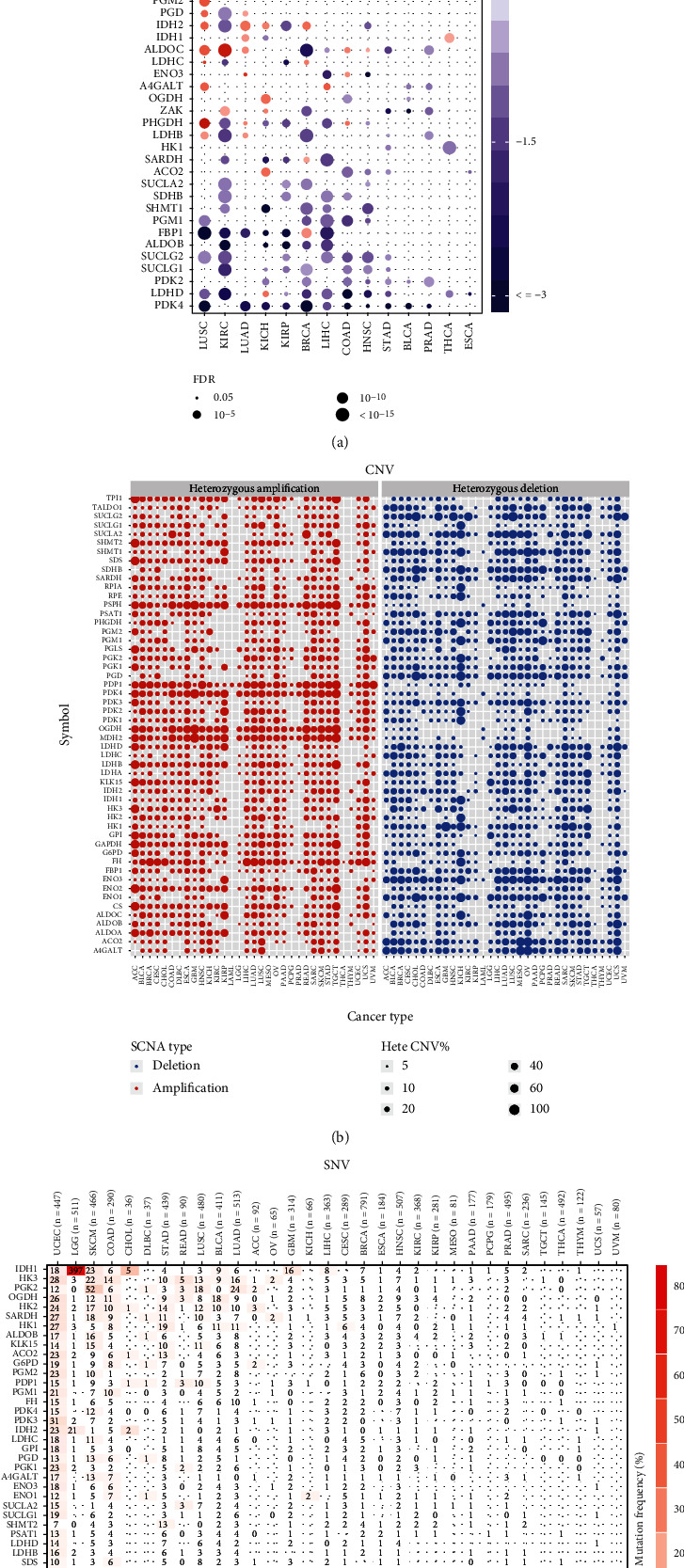
Alterations in the expression genes involved in glycolysis. (a) The gene expression alterations in 14 cancer types. (b) The CNV alteration frequency of genes across cancer types. The left part of each grid shows the amplification frequency, and the right part shows the deletion frequency. (c) The SNV alteration frequency of genes across cancer types.

We searched the literature and identified 53 genes involved in glycolysis. We used these genes to map the glycolysis and tricarboxylic acid cycle pathways (Figure 1). First, we investigated the alteration in the expression of the glycolysis-related genes in 14 cancer types. The results revealed the presence of many differentially expressed glycolysis-related genes in various cancers compared to that in the control samples (Figure 2(a)). We then explored the copy number variation (CNV) alteration frequency for all genes and found that CNV alterations were widespread. The genes with CNV amplifications exhibited a significantly higher expression in cancer tissues than in normal controls (e.g., HK3 and ENO2), while the genes with CNV deletions exhibited significantly lower expression (e.g., ALDOB and PSAT1) (Figure 2(b)). Single nucleotide variants (SNVs) showed that the overall average mutation frequency of all genes was low, especially in ccRCC (0.00–1.09%; Figure 2(c)). Combined with the changes in gene expression, we speculated that CNV alterations may be one of the important mechanisms for modifying gene expression in ccRCC.

**Figure 3:**
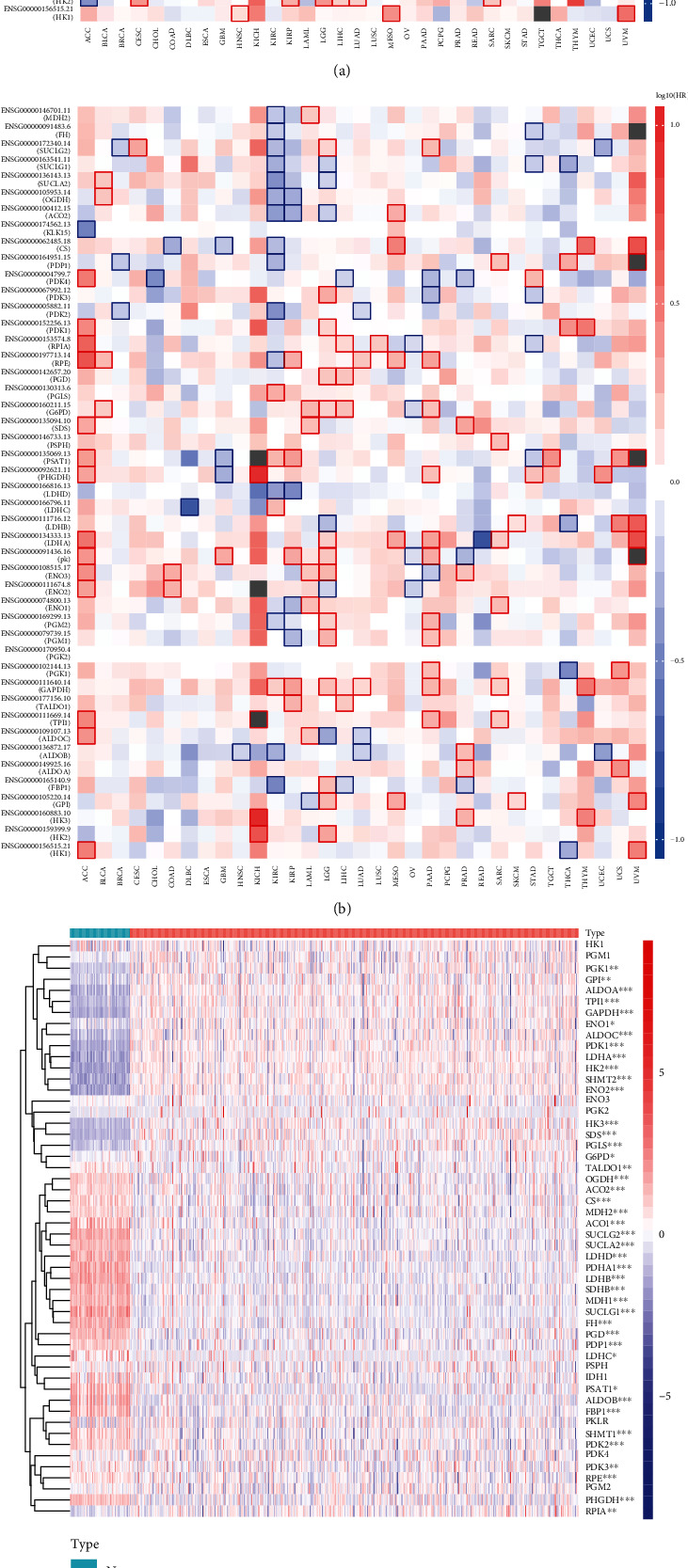
Relationship between gene expression levels and hazard ratio (HR) of ccRCC. (a) The relationship between gene expression levels and overall survival (OS). (b) The relationship between gene expression levels and disease free survival (DFS). (c) The expression levels of 52 genes in 539 ccRCC samples and 72 normal control samples. (d) The expression levels of all genes involved in glycometabolism in ccRCC. ^∗^*p* < 0.05, ^∗∗^*p* < 0.01, and ^∗∗∗^*p* < 0.001.

We first explored the relationship between gene expression levels and the hazard ratio (HR) of ccRCC. The OS and disease-free survival (DFS) of ccRCC patients were obtained from the GEPIA website. The high expression of HK3, ENO2, ENO3, and PSAT1 indicated a short OS and a high risk of developing renal cancer. The high expression of GPI, FBP1, ALDOB, and SUCLA2 indicated longer OS and a low risk of developing renal cancer (Figure 3(a)). The high expression of GAPDH, PSAT1, PGLS, and LDHC indicated a short DFS and a high risk of developing renal cancer. The high expression of FBP1, ALDOB, LDHD, and SUCLA2 indicated a longer DFS and a low risk of developing renal cancer (Figure 3(b)). Next, we compared the expression levels in 539 ccRCC tissues and 72 normal renal tissues in TCGA dataset and obtained 43 differentially expressed glycolysis-related genes. Compared with those in the normal control group, patients with ccRCC exhibited higher expression of PGK1, GPI, ALDOA, TPI1, GAPDH, ENO1, ALDOC, PDK1, LDHA, HK2, SHMT2, ENO2, HK3, SDS, PGLS, G6PD, and PRIA (Figure 3(c)). The expression levels of genes involved in glucose metabolism were consistent with this result (Figure 3(d)). Therefore, we speculated that these 43 differentially expressed glycolysis-related genes may be related to the occurrence of ccRCC.

### 3.2. Gene Coexpression Network Analysis and the Search for Potential Transcriptional Factors

**Figure 4:**
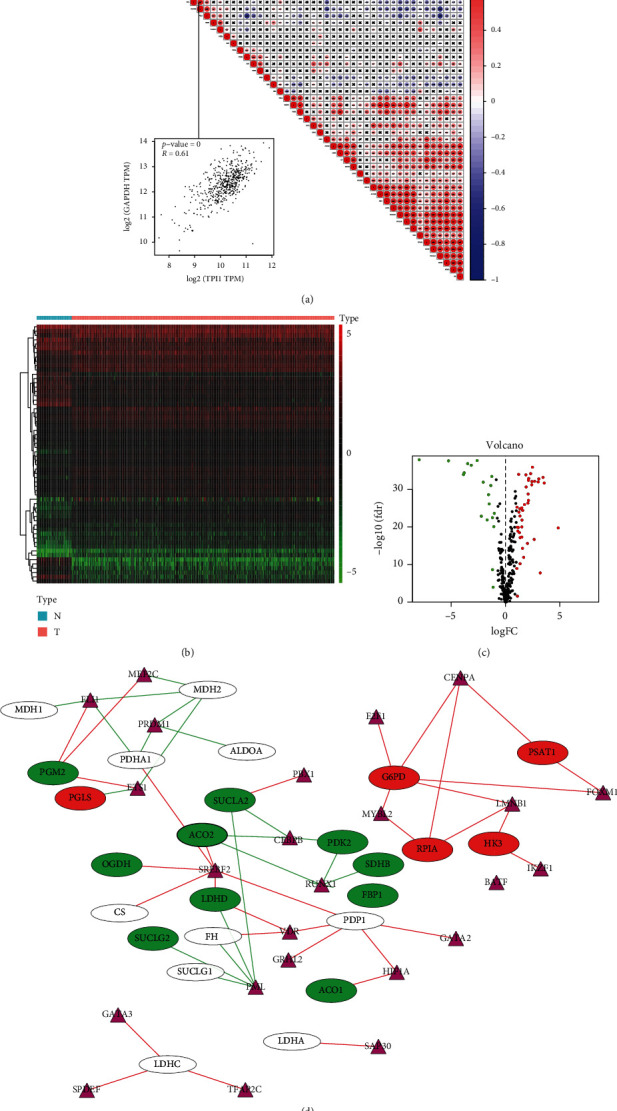
Coexpression network for the glycolysis-related genes and the transcriptional factor network between ccRCC group and normal control group. (a) Coexpression network for glycolysis-related genes and the correlation between GAPDH and TPI1. (b) The expression levels of transcriptional factors in 539 ccRCC samples and 72 normal control samples. (c) Volcano plot of all transcriptional factors; red color represents transcriptional factors with high expression, green color represents transcriptional factors with low expression, and black color represents transcription factors with no differential expression. (d) The transcriptional factor upstream of the main differentially expressed glycolysis-related genes in ccRCC. The purple triangle represents the transcriptional factors, the red color represents upregulated glycolysis-related genes, and the green color represents downregulated glycolysis-related genes.

Coexpression network revealed that glycolysis-related genes exhibited a strong coexpression relationship. We used the GEPIA website to analyze the correlation between GAPDH and TPI1 and found that they were very significant (*R* = 0.61). When the GAPDH gene was upregulated, TPI1 was also most likely upregulated (Figure 4(a)). As shown in Figure 4(b), transcription factors differentially expressed in the cancer and normal control groups were observed in the heat map. We analyzed all the differentially expressed transcriptional factors, and the volcano plot revealed that 41 transcription factors were upregulated and 19 transcription factors were downregulated in ccRCC (*p* < 0.05, log_2_ fold − change > 1) (Figure 4(c)). To investigate transcriptional factors upstream of the main differentially expressed genes in ccRCC, we generated a transcriptional factor network and observed that several core transcriptional factors were associated with the differentially expressed genes namely, FLI1, ETS1, SREBF2, PML, CEBPB, RUNX1, MYBL2, CENPA, FOXM1, and LMNB1 (Figure 4(d)).

### 3.3. Categories Determined by Consensus Clustering Were Closely Correlated to the Clinical Outcomes and Clinicopathological Features

**Figure 5:**
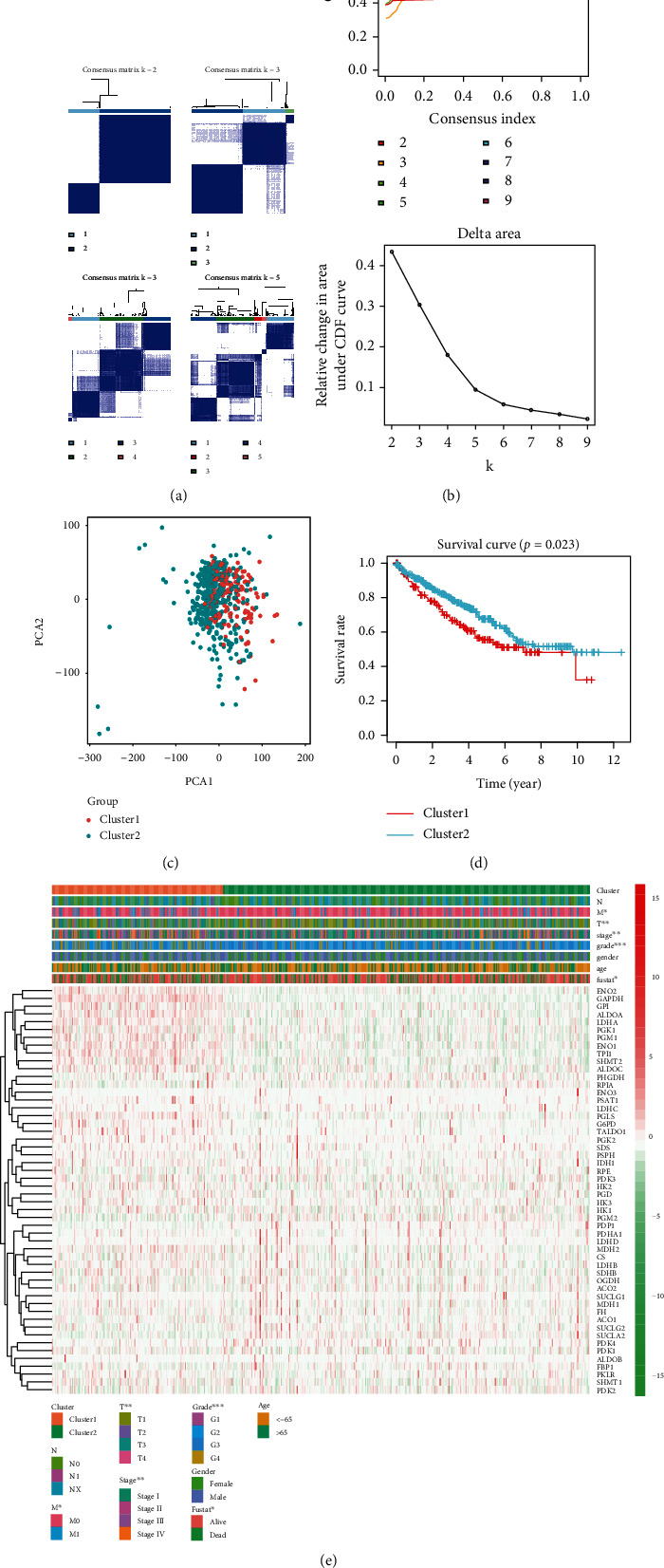
Identification of consensus clusters based on glycolysis-related genes and overall survival of ccRCC in the cluster 1/2 subgroups. (a) Consensus clustering matrix for *K* = 2 − 5. (b) Consensus clustering cumulative distribution function (CDF) for *K* = 2 − 9, relative change in area under the CDF curve for *K* = 2 − 9. (c) PCA of the total genes in TCGA dataset. (d) Kaplan-Meier overall survival (OS) curve for 539 ccRCC patients. (e) Based on the results of this cluster analysis, heat map shows the correlation with clinicopathological characteristics.

ConsensusClusterPlus was used to group 539 cancer tissues. Based on the similarity in the expression of glycolysis-related genes, *k* = 2 had the smallest CDF value; therefore, the cancer tissues were divided into two groups (Figures 5(a) and 5(b)). We analyzed the two subgroups to validate our classification by PCA, and the results showed that both cluster1 and cluster 2 could also be clustered independently (Figure 5(c)). Furthermore, we analyzed the clustering results and OS curves of 539 ccRCC patients and found that the cluster 1 subgroup was associated with a significantly shorter OS than the cluster 2 subgroup (Figure 5(d)). Moreover, we found that most of the glycolysis-related genes were highly expressed in the cluster 1 subgroup. Compared to the cluster 2 subgroup, the cluster 1 subgroup was significantly correlated with a higher stage, higher grade, higher T status, higher M status, and fustat (Figure 5(e)).

### 3.4. Drug Sensitivity and Pathway Analysis

**Figure 6:**
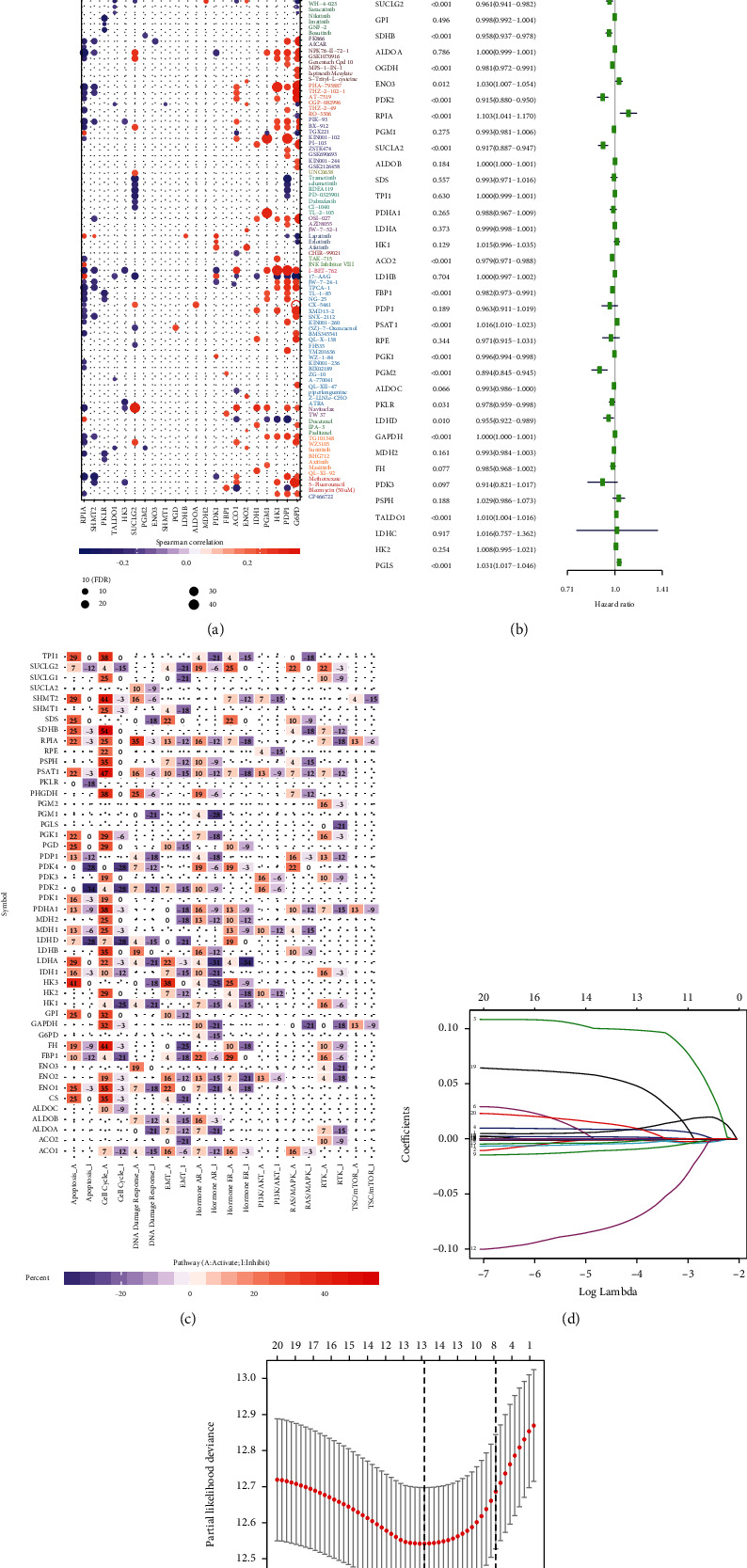
Drug sensitivity analysis and signaling pathway analysis of the glycolysis-related genes and the process of establishing the risk signature. (a) Red indicates that the gene is positively correlated with drug sensitivity. Purple represents that the gene is negatively correlated with drug sensitivity. (b) Univariate Cox regression analysis. (c) Activation or inhibition of different signaling pathways by glycolysis-related genes. (d), (e) LASSO regression analysis and verification.

Drug sensitivity was analyzed using Genomics of Drug Sensitivity in Cancer (GDSC; https://www.cancerrxgene.org/) [25]. The expression of some differentially expressed genes, such as PRIA, SUCLG2, ENO2, and G6PD, was positively correlated with drug sensitivity. The high expression of G6PD was positively correlated with sensitivity to 5-fluorouracil, CX-5461, AT-7519, and PHA-793887, and the high expression of HK3 was negatively correlated with sensitivity to 5-fluorouracil, ATRA, and I-BET-762 (Figure 6(a)). Pathway analysis revealed that PSAT1 and SDHB could activate the cell cycle, RPIA could activate the DNA damage response, and HK3 could activate apoptosis and EMT, while PDK2 could inhibit apoptosis (Figure 6(c)).

### 3.5. Establishment of a Risk Signature and Its Prognostic Value in ccRCC

To explore the prognostic role of differentially expressed glycolysis-related genes in ccRCC, we subjected gene expression data from TCGA to univariate Cox regression analysis. Twenty-five genes were found to have prognostic value with respect to ccRCC (*p* < 0.05) (Figure 6(b)). Among these, IDH1 (HR = 1.022, 95%CI = 1.008–1.037), G6PD (HR = 1.043, 95%CI = 1.028–1.057), HK3 (HR = 1.151, 95%CI = 1.089–1.217), ENO2 (HR = 1.088, 95%CI = 1.004–1.012), ENO3 (HR = 1.030, 95%CI = 1.007–1.054), RPIA (HR = 1.103, 95%CI = 1.041–1.170), PSAT1 (HR = 1.016, 95%CI = 1.010–1.023), GAPDH (HR = 1.000, 95%CI = 1.000–1.001), TALDO1 (HR = 1.010, 95%CI = 1.004–1.016), and PGLS (HR = 1.031, 95%CI = 1.017–1.046)—upon being expressed at high levels—resulted in a short survival duration in ccRCC patients. On the contrary, SHMT1 (HR = 0.971, 95%CI = 0.956–0.987), ENO1 (HR = 0.999, 95%CI = 0.999–1.000), ACO1 (HR = 0.955, 95%CI = 0.921–0.991), PDK4 (HR = 0.997, 95%CI = 0.996–0.999), SUCLG2 (HR = 0.961, 95%CI = 0.941–0.982), SDHB (HR = 0.958, 95%CI = 0.937–0.978), OGDH (HR = 0.981, 95%CI = 0.972–0.991), PDK2 (HR = 0.915, 95%CI = 0.880–0.950), SUCLA2 (HR = 0.917, 95%CI = 0.887–0.947), ACO2 (HR = 0.979, 95%CI = 0.971–0.988), FBP1 (HR = 0.982, 95%CI = 0.973–0.991), PGK1 (HR = 0.996, 95%CI = 0.994–0.998), PGM2 (HR = 0.894, 95%CI = 0.845–0.945), PKLR (HR = 0.978, 95%CI = 0.959–0.998), and LDHD (HR = 0.955, 95%CI = 0.922–0.989)—upon being expressed at high levels—resulted in a longer survival duration in patients with ccRCC.

**Figure 7:**
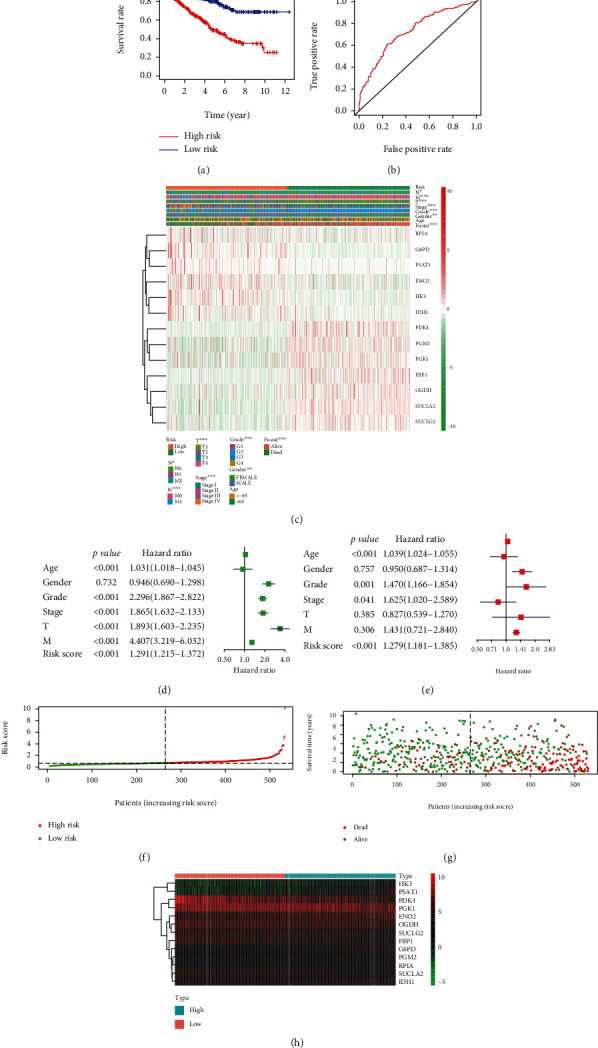
Relationship between the risk scores, clinicopathological factors, and clusters. (a) Kaplan-Meier overall survival (OS) curves based on this risk model. (b) ROC curves showed the predictive ability of the risk signature. (c) The heat map showed the expression levels of the 13 glycolysis-related genes in low- and high-risk ccRCC patients and the correlation analysis with clinicopathological factors. (d) Univariate Cox regression analyses. (e) Multivariate Cox regression analyses. (f) The relationship between risk scores and high risk and low risk. (g) The relationship between risk scores and survival time. (h) The heat map showed the expression levels of the 13 glycolysis-related genes in low- and high-risk ccRCC patients.

Subsequently, we selected 20 genes that were identified as being significant in the univariate Cox analysis (*p* < 0.01) for the LASSO regression analysis. Thirteen genes were selected to generate the risk signature, and the coefficients obtained from the LASSO algorithm were used to calculate the risk score (Figures 6(d) and 6(e)). We then stratified the ccRCC patients into the low-risk and high-risk groups according to the median risk score; the results indicated that patients in the high-risk group had a lower survival rate (Figure 7(a)). ROC curve analysis was performed to predict the risk scores and survival rate of ccRCC patients; the area under the curve (AUC) was found to be 0.731. The results showed that the risk scores could predict the survival rate of patients with ccRCC (Figure 7(b)).

### 3.6. The Risk Signature Was Closely Related to the Clinicopathological Characteristics of ccRCC

We studied the relationship between the 13 selected genes in the high- and low-risk groups—from TCGA—and the pathological characteristics of cancer, including age, stage, grade, T status, M status, and N status. We found that these 13 genes were closely related to the pathological characteristics of cancer. Moreover, compared with patients in the low-risk group, ccRCC patients in the high-risk group exhibited higher expression of RPIA, G6PD, PSAT1, ENO2, HK3, and IDH1 and lower expression of PDK4, PGM2, PGK1, FBP1, OGDH, SUCLA2, and SUCLG2 (Figures 7(c) and 7(h)).

We performed univariate and multivariate Cox regression analyses on TCGA data. Univariate Cox regression analyses indicated that the risk score, age, stage, grade, T status, and M status were all related to OS. As the risk score, age, stage, grade, T status, and M status increased, the risk increased. Multivariate Cox regression analyses indicated that the risk score, age, stage, and grade were independent risk factors (Figures 7(d) and 7(e)). Furthermore, as the risk scores increased, the risk of developing cancer and the number of deaths increased (Figures 7(f) and 7(g)). Based on these results, we conclude that the established risk signature is closely correlated with the clinicopathological characteristics of ccRCC.

## 4. Discussion

Glycolysis is a natural process that occurs in normal tissues under hypoxic conditions. Glycolysis has also been observed in areas of malignant tumors with sufficient oxygen. To enable the proliferation of tumor cells and to enable the formation of a membrane structure, it is necessary to produce a large number of fatty acids, a process that requires aerobic glycolysis, also known as the Warburg effect. This phenomenon is very common in malignant tumors [26–29]. Glycolysis is very important for tumors, and tumor cells exhibit high expression of key enzymes involved in the process of glycolysis to generate more energy. In addition, tumor cells can also produce energy through the fermentation of lactic acid, a product of glycolysis [30]. The Warburg effect and glycolytic genes have been studied in lung cancer, breast cancer, ovarian cancer, gastric cancer, liver cancer, colorectal cancer, esophageal cancer, prostate cancer, bladder cancer, and other tumors [11–19].

A recent study has shown that the Warburg effect in ccRCC is more pronounced than that in other tumors [31]. A report based on metabolic atlas showed that ccRCC is characterized by an increase in the level of metabolites during glycolysis and a decrease in the level of metabolites during oxidative phosphorylation, suggesting that glycolysis is active in ccRCC [21]. Therefore, we chose to study the expression of glycolysis-related genes in ccRCC patients to identify biomarkers that can predict the prognosis of the disease. Unlike many previous studies that may be limited to a single gene [32, 33], our research attempts to further understand the occurrence and development of tumors by studying the entire biological pathway. This new type of research is currently being respected and has made progress [34–36].

Our study included 539 ccRCC tissue samples and 72 normal control tissue samples from the TCGA dataset. We analyzed 53 glycolysis-related genes and identified 43 differentially expressed genes between the ccRCC and normal control groups. In addition, we identified 25 genes related to the prognosis of ccRCC patients using univariate Cox analysis. Finally, we used LASSO regression analysis to construct a risk model consisting of 13 genes: RPIA, G6PD, PSAT1, ENO2, HK3, IDH1, PDK4, PGM2, PGK1, FBP1, OGDH, SUCLA2, and SUCLG2.

ENO2 is a key gene in glycolysis, which catalyzes the dehydration of 2-phosphoglyceric acid to produce phosphoenolpyruvate. It can promote cell growth, upregulate glycolysis-related genes, and activate Akt signaling pathway through phosphorylation of glycogen synthase kinase3*β*, so as to induce cell proliferation and glycolysis [37]. Increased expression of ENO2 has been found in many types of tumors. The high expression of ENO2 in glioma and colorectal cancer is related to glycolysis in the tumor cells [38, 39]. The increased expression of ENO2 promotes glycolysis in gastric cancer cells, which is related to tumor growth and liver metastasis [40]. In terms of Warburg effect in renal cell carcinoma, recent studies proposed that the expression of ENO2 was significantly higher in the tissues and serum of RCC patients [41, 42]. In addition, the increase in serum ENO2 level was related to clinical stage, tumor grade, and disease recurrence; therefore, it is a potential biomarker for the prognosis of RCC [43, 44]. In our study, ENO2 was highly expressed in the tissues of ccRCC patients and played a role in activating the cell cycle, EMT, and PI3K/AKT signaling pathway, which was consistent with our expectations. Meanwhile, the results indicated that ENO2 may promote the growth and invasion of ccRCC cells through glycolysis and that the high expression of ENO2 may be related to the Warburg effect.

Metabolic transfer to glycolysis is related to changes in the signaling pathways involved in energy metabolism. This process involves the ingestion and fermentation of glucose. HK3 is a key gene in glycolysis, and the first step of hexokinase- (HK-) catalyzed glycolysis has been confirmed to be very important in the development of colorectal cancer and melanoma [45]. The expression of glycolytic enzymes, including HK3, was also increased in breast cancer, and HK3 was considered to be the most important gene in pediatric acute lymphoblastic leukemia; it plays an important role in the prognosis of the disease through the glycolytic pathway [46, 47]. The increased expression of HK3 is related to EMT in colorectal cancer, which is involved in the rapid growth and metastasis of colorectal cancer [48]. At present, there are few reports on HK3 in renal cell carcinoma. In our study, HK3 played a role in activating apoptosis and the EMT signaling pathway, which is consistent with previous research results. Based on the expression of HK3 in other tumors, we speculated that the high expression of HK3 in ccRCC was related to the Warburg effect, and HK3 could promote the rapid proliferation, invasion, and metastasis of ccRCC by enhancing apoptosis and epithelial-mesenchymal transition.

FBP1 catalyzes the formation of fructose-6-phosphate from fructose-1,6-diphosphate and water, which plays a key role in gluconeogenesis. FBP1 is a downstream glycoisoenzyme and tumor suppressor that inhibits glycolysis and tumor growth and partially inhibits tumor growth by inhibiting mitotic signal transduction [49, 50]. As a tumor suppressor, decreased expression of FBPT1 in a variety of tumors has been reported in many studies. The low expression of FBP1 promoted the invasion of hepatocellular carcinoma cells through the Warburg effect [51]. When FBP1 was downregulated, glycolysis increased, and the decrease in FBP1 level reprogrammed the metabolism of glioblastoma cells [52]. The expression of FBP1 was downregulated in gastric cancer and gastric cancer cell lines, and this downregulation was related to the Warburg effect in tumors [53]. High expression of FBP1 inhibits the growth, metastasis, and glycolysis of breast cancer [54]. Previous studies have shown that FBP1 could inhibit glycolysis in kidney cancer, and the expression of FBP1was significantly lower in patients with high-grade ccRCC than in patients with low-grade ccRCC [55]. FBP1 could antagonize the glycolytic flux of renal tubular epithelial cells, the assumed cells of ccRCC, thus inhibiting the Warburg effect [56]. In our study, FBP1 was downregulated in the tissues of ccRCC patients, suggesting that glycolysis was enhanced in ccRCC, while gluconeogenesis was decreased, which was consistent with our expectations. In addition, FBP1 plays a role in inhibiting the cell cycle and EMT pathways, and thus, FBP1 had an inhibitory effect on tumor growth, invasion, and metastasis.

These genes are closely related to the pathological characteristics of cancer. According to clinical characteristics, ccRCC patients were divided into the high-risk and low-risk groups. Compared with the low-risk group patients, the high-risk group patients had a higher proportion of RPIA, G6PD, PSAT1, ENO2, HK3, IDH1, and lower proportions of PDK4, PGM2, PGK1, FBP1, OGDH, SUCLA2, and SUCLG2. As the risk score, age, stage, grade, T status, and M status increased, the risk increased. The Kaplan-Meier overall survival curves showed that the high-risk group had a lower survival rate than the low-risk group, which suggested that the predictive model could predict the survival rate and evaluate the prognosis of ccRCC patients.

The results of drug sensitivity analysis suggested that the low expression of ENO2 and HK3 genes and the high expression of PGK1 were positively correlated with drug sensitivity. In addition, some glycolysis-related genes were positively correlated with drug sensitivity, which could be used as potential targets for drug therapy. We performed a transcription factor network for the main differentially expressed genes of ccRCC, and several core transcriptional factors were identified: FLI1, ETS1, SREBF2, PML, CEBPB, RUNX1, MYBL2, CENPA, FOXM1, and LMNB1. The discovery of these transcription factors provides a basis for protein and functional research.

In this study, we first explored the expression of glycolysis-related genes in ccRCC using integrated bioinformatic analysis. We identified key glycolysis-related genes that are differentially expressed in ccRCC, analyzed the effects of these genes on common signaling pathways, and generated a predictive prognostic model for ccRCC. However, there are still some limitations. Our results have not been validated by in vitro experiments, such as quantitative real-time polymerase chain reaction and western blotting. Further studies on human tissue samples are required to validate these results.

## 5. Conclusions

In conclusion, we identified that key glycolysis-related genes are differentially expressed in ccRCC, analyzed the effects of these genes on common signaling pathways, and constructed a predictive model of ccRCC. This predictive model correlated well with the development and progression of ccRCC and showed great diagnostic and prognostic value. This model may serve as a potential prognostic biomarker and drug target for ccRCC.

## Data Availability

The data used to support the findings of this study are available from the corresponding author upon request.

## Abbreviations

CNV: Copy number variations
SNV: Single nucleotide variant
ccRCC: Clear cell renal cell carcinoma
TCGA: The Cancer Genome Atlas
PSAT1: Phosphoserine aminotransferase 1
SDHB: Succinate dehydrogenase complex iron sulfur subunit B
RPIA: Ribose 5-phosphate isomerase A
G6PD: Glucose-6-phosphate dehydrogenase
ENO2: Enolase 2
HK3: Hexokinase 3
IDH1: Isocitrate dehydrogenase 1
PDK4: Pyruvate dehydrogenase kinase 4
PGM2: Phosphoglucomutase 2
PGK1: Phosphoglycerate kinase 1
FBP1: Fructose-bisphosphatase 1
OGDH: Oxoglutarate dehydrogenase
SUCLA2: Succinate-CoA ligase ADP-forming subunit beta
SUCLG2: Succinate-CoA ligase GDP-forming subunit beta
GEPIA: Gene expression profiling interactive analysis
OS: Overall survival
DFS: Disease-free survival.
